# Intellectual property and access to medicines: mapping public attitudes toward pharmaceuticals during the United States-Mexico-Canada Agreement (USMCA) negotiation process

**DOI:** 10.1186/s12992-021-00740-1

**Published:** 2021-08-20

**Authors:** Anna S. Y. Wong, Clarke B. Cole, Jillian C. Kohler

**Affiliations:** 1 World Health Organization Collaborating Centre for Governance, Accountability, and Transparency in the Pharmaceutical Sector, 144 College Street, Toronto, ON M5S 3M2 Canada; 2 University of Toronto Leslie Dan Faculty of Pharmacy, 144 College Street, Toronto, ON M5S 3M2 Canada

**Keywords:** Transparency, Accountability, Pharmaceuticals, Drug industry, Government, Access to medicines, International trade

## Abstract

**Background:**

Transparency and accountability are essential components at all stages of the trade negotiation process. This study evaluates the extent to which these principles were upheld in the United States’ public consultation process during the negotiation of the United States-Mexico-Canada Agreement (USMCA), with respect to public comments about the pharmaceutical sector and access to medicines.

**Results:**

The public consultation process occurred before the start of official negotiations and was overseen by the Office of the United States Trade Representative (USTR). It included both written comments and oral testimony about US trade negotiation objectives. Of the written comments that specifically discussed issues relating to pharmaceuticals, the majority were submitted by private individuals, members of the pharmaceutical industry, and civil society organizations. Nearly all comments submitted by non-industry groups indicated that access to medicines was a priority issue in the renegotiated agreement, with specific reference to price affordability. By contrast, more than 50% of submissions received from members or affiliates of the pharmaceutical industry advocated for strengthened pharmaceutical intellectual property rights, greater regulatory data protections, or both.

This study reveals mixed outcomes with respect to the level of transparency achieved in the US trade negotiation process. Though input from the public at-large was actively solicited, the extent to which these comments were considered in the content of the final agreement is unclear. A preliminary comparison of the analyzed comments with the USTR’s final negotiating objectives and the final text of the USMCA shows that several provisions that were advanced exclusively by the pharmaceutical industry and ultimately adopted in the final agreement were opposed by the majority of non-industry stakeholders.

**Conclusions:**

Negotiators could increase public transparency when choosing to advance one competing trade objective over another by actively providing the public with clear rationales for their negotiation positions, as well as details on how public comments are taken into account to form these rationales. Without greater clarity on these aspects, the public consultation process risks appearing to serve as a cursory government mechanism, lacking in accountability and undermining public trust in both the trade negotiation process and its outcomes.

**Supplementary Information:**

The online version contains supplementary material available at 10.1186/s12992-021-00740-1.

## Introduction: background on trade agreements and the transparency deficit

In trade negotiations, governments are faced with managing the competing interests of different sectors and stakeholders within their domestic populations. This often includes balancing national economic imperatives with policies seeking to improve health outcomes, such as expanding access to essential medicines – a health systems component critical to upholding the human right to health [1]. Conflicting government priorities are particularly apparent during trade negotiations, where gains in one area often come at the cost of concessions in others. Amidst increasingly polarized stakeholder interests with respect to pharmaceutical innovation, drug pricing, and equitable access to medicines, it is therefore vital to ensure that the trade positions adopted by government negotiators accurately reflect the interests of the citizens they represent. This tension forms the backdrop for this research paper.

Transparency and accountability are integral to the trade negotiation process. Policies that embrace transparency ensure that information about the plans, processes, and actions of government negotiators are made publicly available, easily accessible, and understandable to citizens [2]. Increased transparency can in turn promote policies of public accountability [3, 4], such as sanction-backed requirements that agencies involved in trade negotiations provide rationales for their negotiation decisions [5]. Taken together, transparency and accountability provide safeguards to promote government decision-making that maximizes public engagement with the development of national trade agendas and the negotiation of trade agreements.

Multilateral trade agreements under the World Trade Organization (WTO) include a number of transparency obligations. However, oversight of bilateral and regional trade agreements made by WTO member states is limited to the WTO Transparency Mechanism, which merely requires countries to “report trade measures to the relevant WTO body if the measures might have an effect on other members” [6]. Beyond this, the level of transparency achieved in a given trade negotiation and its resulting agreement depends on the self-ascribed standards adopted by the participating states. This transparency deficit has been noted by several civil society organizations, such as Open Government Partnership and Transparency International [7, 8], and is exemplified through practices including closed-door negotiations, a lack of public access to relevant negotiation documents, and limited windows during which the public is permitted to provide feedback on trade proposals [8]. Such practices have a significant effect on the negotiation process. When public access to information and decision-makers is limited, actors with the resources to form special connections with decision-makers are disproportionately advantaged to advocate for their preferred policy outcomes. This dynamic can undermine the public legitimacy of trade agreements if the agreements are subsequently perceived to not represent the interests of the public at large.

There is a lack of research that systematically explores the degree of transparency and accountability embedded in the trade negotiation process. While recent studies have analyzed the risks new bilateral and regional trade agreements pose to public health policy [9–13, 35], the study of the “processes and factors that influence the implementation of trade treaties” [14] and “the political and financial pressure exercised by interest groups and external, non-trade state pressure to shape trade treaties” [14] remains underdeveloped. Without a greater understanding of the extent to which the trade negotiation process facilitates public participation, it is difficult to gauge how accurately the actual content of free trade agreements reflect the public interest.

This paper aims to contribute to transparency and accountability in international trade negotiations by evaluating the United States’ public consultation process during the renegotiation of the North American Free Trade Agreement (NAFTA), now predominantly referred to as the United States-Mexico-Canada Agreement (USMCA). In this paper, we map the USMCA negotiation process and analyze the public comments relating to medicines and pharmaceuticals submitted during the United States’ (US) consultation period. In doing so, the policy priorities advanced by the various classes of USMCA stakeholders relating to health, access to medicines, and international trade are identified and evaluated.

## Methods

Since most aspects of trade negotiations are held under conditions of strict confidentiality, the public consultation process provides the primary window through which the public can actively engage with the development of trade policy. The US was selected as the country for analysis based on the immediate and robust availability of its public consultation documents. An analysis of Mexico’s public consultation process was excluded because its public consultation was limited to industry and commercial stakeholders rather than the public at large [15, 16]. An analysis of Canada’s public consultation process was excluded because of a lack of public access to its consultation documents at the time of data collection [17].

A qualitative research study focussing on publicly available documents was completed to map the US negotiation rounds of the USMCA. Government and other administrative actors directly involved in the trade negotiation process were identified, with the mechanisms for filling their positions (e.g., presidential appointment, public nomination) recorded. Then, opportunities for the public to receive information about the trade negotiations and to provide feedback about the agreement’s proposed provisions were noted. While records of both written comments and oral testimonies made by stakeholders are publicly available, oral testimonies were delivered by a subset of stakeholders that also submitted written comments. Thus, only written comments were selected for analysis due to their more comprehensive representation of the total feedback submitted by members of the public. Finally, the written comments submitted by stakeholders during the public consultation process were systematically queried for language related to pharmaceuticals.

### Public comments: search strategy

Regulations.gov is the US government’s online platform that enables members of the public to find, read, and comment on officially issued federal regulations and related documents [18]. During the public consultation process, written comments were submitted by stakeholders to the US Trade Representative (USTR) through the Regulations.gov platform at Docket USTR-2017-0006 *Requests for Comments: Negotiation Objectives Regarding Modernization of North American Free Trade Agreement with Canada and Mexico* (“the Docket”). The search tool embedded in the ‘Comments’ tab of the Docket was used to query the set of publicly available comments submitted during the public consultation period with the search terms *pharma, pharmaceutical, medicine, medical, drug,* and *health* to identify an initial pool of comments for analysis. These search terms were developed based on the search engine’s functionality, which, for example, is not sensitive to wildcard (*) searches. The resulting comments were then downloaded from the Docket. Comments that were identified multiple times by different search terms (i.e., comments with identical Docket IDs) were only recorded once.

**Fig. 1:**
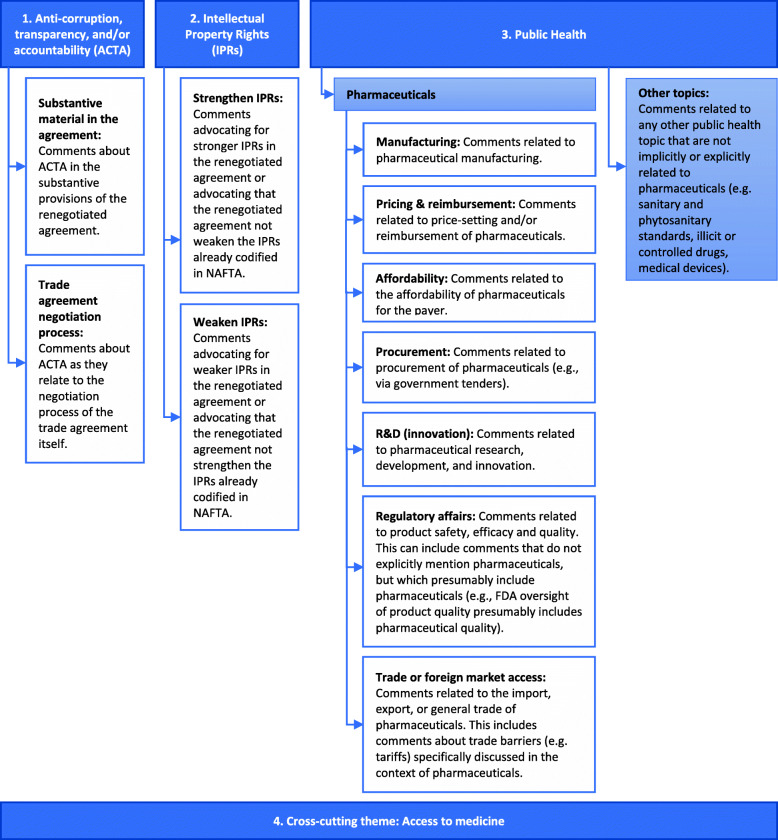
Node Tree. The following classifications were used for qualitative analysis of the submitted comments. Nodes are not mutually exclusive. NAFTA = North America Free Trade Agreement, FDA = US Food and Drug Administration

To facilitate qualitative analysis, a hierarchical coding tree (‘node tree’) was constructed in two iterative rounds of review between the authors based on a sampling of 20 downloaded comments. The downloaded comments were annotated based on this tree (see Fig. 1) using qualitative data analysis software NVivo. Each comment was also assigned one of six submitter identity classifications, defined based on affiliations self-reported in the ‘Submitter Information’ section of each comment (see Table 1). Duplicative comments submitted a second time by the same organization (i.e., identical comments with different Docket IDs), comments not related to pharmaceuticals for human use (e.g., comments related to pharmaceutical use in animals), and comments that mentioned the pharmaceutical sector for the sole purpose of illustrating the size of another industry were excluded from analysis.

**Table 1:** Submitter identity classifications. The following shows the categories of stakeholders that submitted comments to the Docket, and a description of each stakeholder type

Submitter Type	Description
**Academia**	Submitter is affiliated with or writing on behalf of a university, college, research centre, or think tank.
**Civil Society**	Submitter is affiliated with or writing on behalf of a civil society organization (e.g., non-governmental organization, non-profit, advocacy group). Submitter may also be a representative of a civil society group submitting a comment on behalf of its members.
**Government**	Submitter is a member of elected government or a government worker writing on behalf of their employing office.
**Individual**	Submitter is unaffiliated with any group or organization.
**Industry**	Submitter is writing on behalf of a company in the pharmaceutical industry (including both originator and generic manufacturers), a pharmaceutical industry trade association, or a broad multi-industry coalition whose members include companies in the pharmaceutical industry.
**Professional Association**	Submitter is writing on behalf of a professional association (e.g., trade unions).

## Results

The following section outlines the process by which the US government negotiated the USMCA, including the timeline, involved stakeholders, and mechanisms through which the public was consulted. It also presents the major findings from the qualitative analysis of the comments submitted to the Docket, with respect to the number of submissions, submitter identities, and substantive policy themes.

### Overview of the USMCA negotiations

Trade negotiations for the USMCA formally began on 16 August 2016 and were concluded on 30 September 2018 after seven rounds of negotiations between the US, Mexico and Canada [19, 20]. On 30 November 2018, the USMCA was signed by all three parties at the G20 Buenos Aires Summit. On 29 January 2020, it was signed into law by the United States. It was ratified by Mexico and Canada on 19 June 2019 and 3 April 2020 respectively. On 1 July 2020, it formally entered into force in all three member states [21].

### Government and administrative actors involved in the USMCA negotiations

**Fig. 2:**
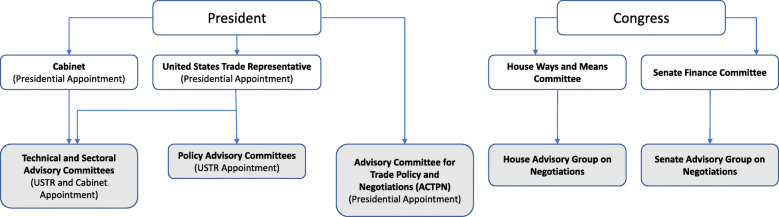
Government stakeholders involved in US trade negotiations. Trade committees and advisory groups are highlighted in grey

The US trade negotiation process involves internal consultations between various groups and committees, including the USTR, the Advisory Committee for Trade Policy and Negotiations (ACTPN), Policy Advisory Committees, and Technical and Sectoral Advisory Committees (Fig. 2). Political appointees are members of the USTR, ACTPN, and Policy Advisory Committees [22, 23]. Committee experts, nominated publicly through the Federal Register, are appointed by the USTR in conjunction with the Cabinet and sit on twenty-two Technical and Sectoral Advisory Committees [22]. Congressional committees and groups related to trade negotiations, as well as the Policy Advisory Committees, are continuously consulted throughout the trade negotiation process per USTR guidelines under the *Trade Promotion Authority Act*, *2015* [19, 22]. Before announcing the Administration’s intent to renegotiate NAFTA, the Trump Administration also held over three months of consultation meetings with the USTR, members of the House Ways and Means Committee, Senate Finance Committee, and Congressional Advisory Groups on Negotiations [19, 20].

### USMCA public consultation process

Between 23 May – 14 June 2017, prior to the start of official negotiations with Canada and Mexico, the USTR solicited input from the US public about its negotiation objectives. Publications in the Federal Register, press releases from the USTR website, and tweets from the USTR official Twitter account (@USTradeRep) directed members of the public to the Regulations.gov website to submit comments [24–28]. At the end of the public consultation period, the USTR received 12,460 comments [25].

The USTR also held three days of public hearings at the U.S. International Trade Commission in Washington, D.C. to solicit input from over 140 stakeholders [29]. Stakeholders wishing to testify were required to provide written notification of their intent to testify, however the process for selecting the testifying participants remains unclear. A list of the participating stakeholders, the organizations they represented, and video recordings and transcripts of the hearings were made publicly available on the USTR website [29].

On 17 July 2017, the USTR released its NAFTA Negotiation Objectives [30]. These objectives reflected the input received by the USTR throughout its consultations with Congress, its advisory committees, and the public. An Updated NAFTA Negotiation Objectives document was released by the USTR on 17 November, 2017 [31]. No further official opportunities were provided to the public to provide additional input on the USMCA negotiations. See Table 2 for an overview of the timeline of the US trade negotiation process.

**Table 2:**
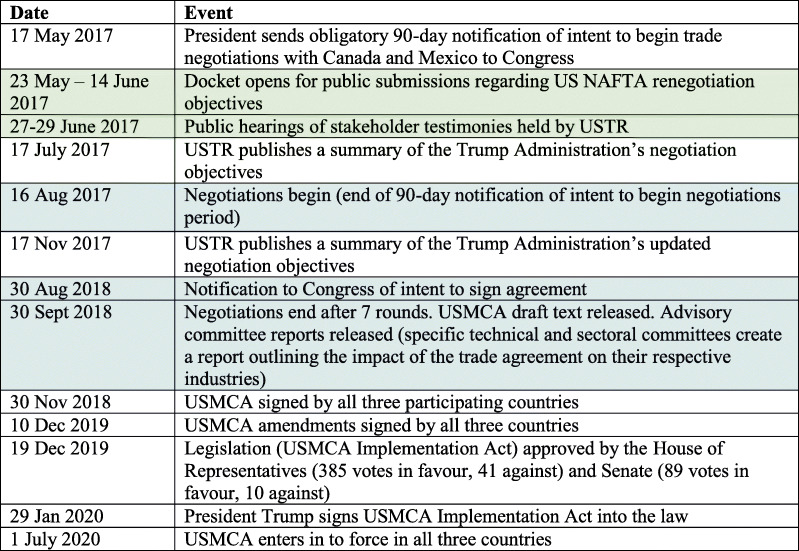
Timeline of US trade negotiation process for USMCA. Opportunities for public input are highlighted in green and negotiation rounds are highlighted in blue

### Analysis of public comments submitted to the docket

#### Number of submissions

**Fig. 3:**
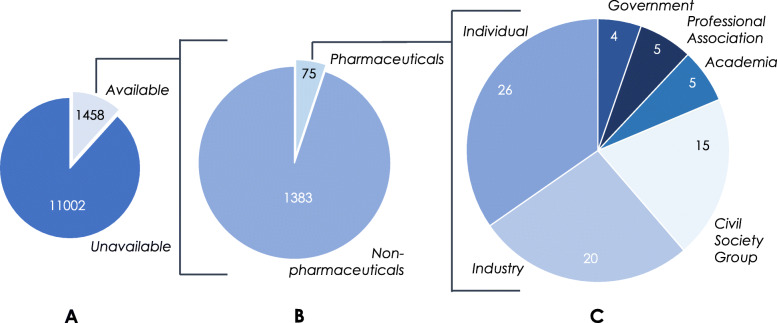
**A** Comments publicly available in the USTR-2017-0006 Docket. **B** Comments related to pharmaceuticals for human use. **C** Classes of stakeholders that submitted pharmaceuticals-related comments

12,460 comments were submitted by stakeholders during the USTR-hosted public consultation process for the negotiation of the USMCA. Of these submissions, 1458 (11.7%) were publicly available on the ‘Comments’ tab of the Docket (Fig. 3A). Additional notes in the Docket state that “Agencies review all submissions, however some agencies may choose to redact, or withhold certain submissions (or portions thereof) such as those containing private or proprietary information, inappropriate language, or a duplicate/near duplicate examples of a mass-mail campaign,” [25] and a Supported & Related Material document notes that over 10,500 additional submitted comments were removed from the ‘Comments’ tab because they were duplicates or near duplicates of a single mass-mail campaign [32]. A copy of this mass-mail submission, as well as five other mass-mail submissions, was provided in the Supported & Related Material section of the Docket [33]. The mass-mail campaign comment submitted 10,530 times to the Docket did not contain any language related to pharmaceuticals or medicines [32]. Another mass-mail campaign submission, containing 12 duplicate or near duplicate submissions, included language related to pharmaceuticals but was excluded from analysis.

Of the 1458 submissions available on the ‘Comments’ tab of the Docket, 288 unique comments contained at least one of the search terms *pharma, pharmaceutical, medicine, medical, drug,* and *health.* Following the exclusion of duplicative comments, comments not pertaining to pharmaceuticals for human use, and comments that exclusively mentioned the pharmaceutical sector to illustrate the size of a different industry, 75 comments were included for analysis (Fig. 3B).

#### Search term frequency

Of the 75 included submissions, 47 contained the search term *health*, 44 contained the search term *pharmaceutical*, 40 contained the search term *medicine*, 29 contained the search term *drug*, 18 contained the search term *medical*, and 7 contained the search term *pharma*. 25 submissions contained a single search term, whereas 50 submissions contained two or more search terms.

#### Submitter identity

In total, 26 comments were received from unaffiliated private individuals, 20 comments were received from representatives or members of the pharmaceutical industry (including Pharmaceutical Research and Manufacturers of America (PhRMA), Biotechnology Innovation Organization (BIO), and the US Chamber of Commerce), 15 comments were received from civil society organizations (including Citizen’s Trade Campaign, Knowledge Ecology International, and People of Faith for Access to Medicines), 5 comments were received from individuals affiliated with academic institutions or think tanks, 4 comments were received from members of government or civil servants writing on behalf of their employing government office (all elected government members who submitted comments were Democrats), and 5 comments were received from professional associations (Fig. 3C). All submitters contributed one single comment each, except Knowledge Ecology International and the American Federation of Labor and Congress of Industrial Organizations, which each submitted two different comments.

#### Prominent themes

**Fig. 4:**
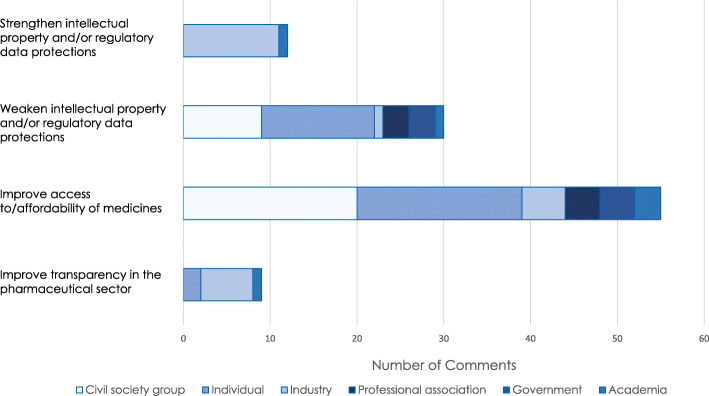
Stakeholder trade priorities in the pharmaceutical sector

Overall, issues of intellectual property and other pharmaceuticals-related exclusivities, access to medicines, and pharmaceutical sector transparency emerged as major stakeholder areas of interest (Fig. 4).

No stakeholders that were ascribed a civil society, government, individual, or professional association identity expressed the opinion that intellectual property rights (IPRs) for pharmaceuticals should increase, with 51% expressly advocating for trade policies that would weaken pharmaceutical IPRs or prevent them from expanding. These included submissions that advocated for the complete elimination of NAFTA’s existing IP protections, the expanded use of TRIPS flexibilities such as compulsory licensing and parallel importation, and the rejection of expanding TRIPS-plus measures that had previously been advanced by the United States during negotiations of the Trans-Pacific Partnership Agreement. In contrast, over half of the submissions in the industry category advocated for either increased IPRs or greater regulatory data protection provisions for pharmaceuticals. In particular, industry submissions predominantly advocated for the harmonization of Canada and Mexico’s IPR and regulatory data policies with that of the United States, including the adoption of strengthened patent linkage provisions to prevent the market entry of allegedly infringing generics and 12-year regulatory data protection periods.

Almost all stakeholders that were ascribed an academic, civil society, government, individual, or professional association identity indicated that access to and/or the affordability of medicines was a priority issue area for pharmaceuticals-related provisions in the renegotiated agreement. Most expressed broad concern about this topic, however some suggested specific policy solutions, such as incorporating trade clauses permitting pharmaceutical price regulation or the importation of medicines from Canada. Of note, only submissions from government stakeholders specifically emphasised the need for a renegotiated agreement to include provisions that enable the US public health system to actively negotiate for lower drug prices. The majority of industry stakeholders indicated that incentivizing pharmaceutical innovation was their priority trade issue. However, some submissions from industry stakeholders also appealed to a rationale of increased access to medicines or lower medicines prices to advocate for stronger of IPRs and/or regulatory data protection terms. For example:

*“National laws, regulations or judicial decisions that prohibit patents on certain types of biopharmaceutical inventions or impose additional or heightened patentability criteria restrict patient access to valuable new medicines and undermine investment in future treatments and cures”* (USTR-2017-0006-0855, Pharmaceutical Research and Manufacturers of America).

*“providing data protection for Rx-to-OTC switches in Canada will lower the cost of medicines, lower health care costs more generally, increase access to medicines, increase market access for health care companies, and bring Canadian intellectual property law up to the standard in United States law.”* (USTR-2017-0006-0913, Consumer Healthcare Products Association).

Of the submissions that discussed agreement provisions related to transparency, more than half were submitted by industry stakeholders. Of these, all but two submissions emphasised the importance of predictability and transparency in the pharmaceutical IP, regulatory, pricing, and reimbursement spaces to facilitate greater innovation, with some explicitly advocating for the inclusion of provisions that would enable greater stakeholder participation in the development of rules and regulations for the biopharmaceutical sector. The remaining two submissions advocated for the inclusion of provisions that would improve pharmaceutical patent transparency to better facilitate the development of generic medicines. These were submitted by the generic industry group Association for Accessible Medicines and the broad multi-industry group United States Council for International Business. Only two comments advocated for increased transparency in the negotiation process itself, and both were submitted by non-industry actors.

For a detailed analysis of comments by submitter identity, see supplemental materials.

## Discussion

### Public consultation process

The process employed by the US during the USMCA negotiations shows some evidence of including transparency mechanisms: opportunities for public input were clearly described in publicly available documents, the public could contribute through both written submissions or oral testimony, and the contents of all submissions were entered into the public record. Furthermore, as many of the government actors involved in the agenda-setting process were elected officials or political appointees, there exists a built-in democratic incentive for accountability to members of the voting public. However, a robust understanding of the extent to which the public’s interest was truly taken into consideration remains incomplete despite these measures.

First, while public stakeholders were notified about the consultation period through several channels, it is difficult to gauge the extent to which the public at large was generally *aware of* and *understood how to navigate* the consultation process. For example, if those who submitted comments to the Docket were drawn from a limited pool of the public that already engages frequently with the federal US public consultation process, this would lead submitted comments to only represent a subset of the perspectives represented by the public at large with respect to trade and health.

Second, while anyone could submit a comment to the Docket, the process by which stakeholders were selected to testify in-person after requesting to participate in the public hearings is unclear. This lack of transparency risks undermining public trust in the consultation process itself, potentially rendering it vulnerable to criticism that the views ultimately adopted by trade negotiators disproportionately reflected those advanced by the individuals and groups with the specialized knowledge and resources required to secure participation in the public hearings.

Finally, since the public consultation period uniquely took place before the trade negotiations officially began, there were no opportunities for the public to provide feedback on the iterative draft agreements made between each round of negotiations. Without such an opportunity, the value of the public consultation process in ensuring that the views and interests of the public-at large are actually brought to the negotiation table is limited; stakeholders are unable to discern whether negotiators advanced their particular positions but yielded as a compromise to make gains in other areas of the agreement, or whether negotiators did not advance their particular positions at all. Even if opportunities for the public to provide further input between draft texts of an agreement are not established, the US government can improve its accountability to the public by actively reporting how stakeholder feedback is taken into account during negotiation rounds.

### Public submissions to the comment docket

Excluding duplicates, a relatively small proportion (5%, or 75 out of 1458) of stakeholder comments were related to pharmaceuticals for human use. This may reflect a low level of public interest or knowledge in these topics relative to other sectoral trade issues. Of the comments included for analysis, the high number of comments submitted by individuals and the pharmaceutical sector indicates a proportionately higher level of interest in pharmaceuticals and access to medicines by these groups compared to the others.

Overall, the comments submitted by the pharmaceutical industry tended to advocate for policies most likely to be commercially beneficial to them, including improved market access and greater IP protections. In select cases where access to medicines was mentioned by members of the pharmaceutical industry, it was used as a rationale to promote trade provisions that were also presented as commercially beneficial. In contrast, non-industry stakeholder groups were primarily interested in promoting access to medicines as an end in itself, framed in the context of personal health outcomes, the integrity of the health system as a whole, and/or the affordability of medicines.

Of the comments submitted by industry stakeholders that discussed provisions within the USMCA related to transparency, most focused on reducing the costs associated with market uncertainty by advocating for improved predictability in pharmaceutical regulation, IP, pricing, and reimbursement in Canada and Mexico. An emphasis on promoting Canadian and Mexican harmonization with the existing US system of IP law and pharmaceutical regulatory affairs suggests a perception by US industry members that legal and regulatory differences between these countries serve as a significant cross-border market barrier. Equally, the industry comments that advocated for increasing transparency in the development of the biopharmaceutical sector’s rules and regulations indicate an industry interest in asserting greater influence over the pharmaceutical regulatory system through participation and consultation in the rule-making process.

A preliminary comparison of the analyzed comments and the final text of the USMCA shows that several provisions that were advanced exclusively by the pharmaceutical industry – and opposed by the majority of comments submitted by non-industry stakeholders – were included in the final agreement. These include provisions for patent term restoration mechanisms, 5-year regulatory data protection minimums for new pharmaceutical products, and 10-year regulatory exclusivities for biologics [34]. Notably, such provisions significantly overlap with several similar provisions initially proposed during the negotiation of the Trans-Pacific Partnership Agreement (now in force as the Comprehensive and Progressive Agreement for Trans-Pacific Partnership, or CPTPP), but that were suspended following the withdrawal of the US from the Agreement. This suggests that these terms may have been strongly advocated for inclusion in the USMCA by US negotiators, despite only being supported by approximately one quarter (26%) of stakeholders that submitted comments about pharmaceuticals. This is supported by the USTR’s final negotiating objectives, which recognized that the Doha Declaration on IP and public health should be respected but emphasized the importance of securing provisions that could increase market access for US products and reflect a standard of IP protection similar to that found in the US – a standard generally considered stronger than those in Canada and Mexico [31].

Support for stronger intellectual property provisions in trade agreements has been historically presented as a dichotomy, with proponents stemming from the pharmaceutical industry and opponents from civil society public health advocates. Though nearly three decades has passed since the original implementation of Agreement on Trade-Related Aspects of Intellectual Property Rights (TRIPS), the results of this study provide empirical evidence indicating that this dichotomy continues to characterize the trade and health landscape.

## Conclusions

The impact of trade agreements on access to medicines remains a pressing area of global health policy. Incorporating mechanisms that advance transparency and accountability in the negotiation of trade agreements is critical to ensuring that trade policies do not undermine the public good, including improving populations’ access to medicines. The examination of the US public consultation process during the USMCA negotiations, through the lens of pharmaceuticals and access to medicines, reveals mixed outcomes with respect to the level of transparency achieved in the US trade negotiation process. Though input from the public at-large was actively solicited, the extent to which these comments were considered in the content of the final agreement is unclear, particularly given that industry and non-industry stakeholders advocated for largely incompatible pharmaceuticals-related trade policies. Negotiators could increase public transparency when choosing to advance one competing trade objective over another by actively providing the public with clear negotiating position rationales. Without greater clarity on these aspects, the public consultation process risks appearing to serve as a cursory government mechanism, lacking in accountability and undermining public trust in both the trade negotiation process and its outcomes.

## Supplementary Information

**Additional file 1.**

## Data Availability

All comments submitted to Docket USTR-2017-0006 are available at Regulations.gov. The set of comments analysed in this study is available from the corresponding author on reasonable request.
