# Supplementary material for: Intellectual property and access to medicines: mapping public attitudes toward pharmaceuticals during the United States-Mexico-Canada Agreement (USMCA) negotiation process
Source: Global Health. 2021 Aug 20;17:92. doi: 10.1186/s12992-021-00740-1 (PMC8379891; doi:10.1186/s12992-021-00740-1)
Supplement: Supplementary file 1 — Additional file 1. [file 12992_2021_740_MOESM1_ESM.docx]

**Supplementary Material: Detailed Results by Submitter Identity**

| Submitter Identity | Thematic topics | Details |
| --- | --- | --- |
| Academia (n=5)  Professor (UCSF), Postdoctoral Scholar (UCSF),  PhD Candidate (Johns Hopkins), Think tank (Property Rights Alliance), Research center (Mexican Institute for Competitiveness) | 3 submissions addressed issues of **access to medicines**, either raising it as a general concern in the renegotiation process or actively advocating for **weakened pharmaceutical IPRs.** | 2 submissions commented generally, noting that trade agreements often have the result of limiting access to essential medicines.  1 submission advocated for the elimination of IP provisions related to pharmaceuticals in the renegotiated agreement + the increased use of trade flexibilities to promote access to affordable medicines. |
|  | 1 submission took the position that pharmaceuticals **IPRs should be strengthened** (re: regulatory data protection) to incentivize innovation in the sector | Submission (from a think tank) advocated for 12 years of regulatory data protection for biopharmaceuticals + a wide scope for pharmaceutical patentability to incentivize innovation |
|  | 1 submission raised a concern about the **quality** of traded medicines | Submission was concerned about ensuring that illegal or “cheap synthetic” drugs were not traded |
| Civil society (n=15)  Citizens Trade Campaign, Public Citizen, Jubilee USA Network, Center for Policy Analysis on Trade and Health (CPATH), Knowledge Ecology International (x2), Americans for Democratic Action, Electronic Frontier Foundation, US Conference of Catholic Bishops, People of Faith for Access to Medicines, Columban Center for Advocacy and Outreach, The People’s Lobby, NETWORK Lobby for Catholic Social Justice, Health Alliance International, Trade Justice New York Metro | 13 submissions broadly sought to **weaken pharmaceutical IPRs** in the renegotiated NAFTA. All but one appealed to a rationale rooted in increasing access to medicines/lowering the cost of medicines, with the outlier arguing that (1) including IP rules in trade agreements prevents the US from having the flexibility to modernize its domestic IP rules when it wants, and (2) given the opposition to the inclusion of IPRs re: pharmaceuticals, insisting on their inclusion would require the US to make concessions on other aspects of the trade agreement.  0 submissions sought to **increase pharmaceutical IPRs** | 7 submissions advocated for the complete elimination of NAFTA’s existing IP protections – either generally or specifically for pharmaceuticals.  5 submissions urged that pharmaceutical IPRs not be expanded in the renegotiated agreement. Of these comments, 3 submissions specifically urged that TRIPS+ measures from the TPP not be included in the renegotiated agreement  4 submissions advocated for the general adoption of policies that weakened pharmaceutical IPRs. These included the expanded use of TRIPS flexibilities (ex. compulsory licensing and parallel importation)  1 submission advocated for the importance of balancing access to medicines with protecting IPRs |
|  | 8 submissions broadly expressed a displeasure with the existing NAFTA agreement and a desire for the renegotiated agreement to increase **access to medicines** and improve the **affordability of medicines** | Submissions commented generally, raising issue with high drug prices, how NAFTA contributed to a significant increase in the cost of medicines in the US (to the benefit of corporations but not the public), and the importance of ensuring that a renegotiated agreement promotes pharmaceutical competition and does not undermine access to affordable medicines (including generics). |
|  | 3 submissions **opposed ISDS** because it is a barrier to drug pricing regulation | Submissions opposed the use of ISDS on the grounds that it prevents countries from pursuing policies that regulate drug prices/promote access to medicines |
|  | 3 submissions addressed issues of **ACTA** | 2 submissions expressed concern that the NAFTA renegotiations would be a repeat of the TPP, resulting in a ‘rigged’ deal that prioritizes corporate interests and protects pharmaceuticals IPRs, resulting in higher consumer prices. Of these submissions, 1 urged for greater transparency and public participation in the renegotiation process  1 submission advocated for the inclusion of a transparency chapter in the renegotiated agreement, including standards for reporting accurate drug R&D costs and clinical trials data |
| Government (n=4)  Congresswoman Barbara Lee, Congressional Progressive Caucus, Congressman Peter DeFazio, Members of Congress (Rosa De Lauro, Peter DeFazio, Marcy Kaptur, Barbara Lee, Daniel Lipinski, Richard Nolan, Mark Pocan, Tim Ryan, Robert Scott, Paul Tonko) | All submissions concerned about finding strategies to **lower drug prices**, with 3 submissions broadly seeking to lower drug prices by **weakening pharmaceutical IPRs** – either by preventing their expansion or completely eliminating from the agreement | 2 submissions acknowledged the importance of balancing IPRs with access to medicines, but urged that TRIPS+ measures not be adopted  1 submission argued for the complete elimination of pharmaceutical IPRs from the new agreement. |
|  | 2 submissions emphasised the importance of maintaining a US ability to **negotiate for lower drug prices** | Submissions urged that the new agreement ensure that the US maintain an ability to negotiate for lower drug prices |
| Individual (n=26) | 9 submissions broadly sought to **weaken pharmaceutical IPRs** in the renegotiated NAFTA. All appealed to a rationale of lowering drug prices. | 6 submissions advocated for the complete elimination of NAFTA’s existing IP protections for pharmaceuticals.  3 submissions advocated for the removal of IPR provisions that allow pharmaceutical companies to continually extend their monopoly provisions. |
|  | 9 submissions addressed general issues of **access to affordable medicines**. | 7 submissions expressed frustration with the perceived prioritization of corporate profit over public access to affordable medicines.  2 submissions commented generally, urging the renegotiated agreement to increase access to medicines for the public. |
|  | 7 submissions urged the US to **regulate pharmaceutical prices** | Submissions urged the US to include provisions that enable the government to regulate the price of pharmaceuticals |
|  | 2 submissions discussed issues of **ACTA**. | 1 submission advocated for a transparent and democratic agreement that prioritized citizens over corporations  1 submission advocated for increased transparency in product labeling for pharmaceutical products (re: genetically modified biologics) |
|  | 2 submissions emphasised the importance of ensuring that **imported/exported** pharmaceuticals were subject to **safety and quality** inspections | Submissions expressed concern with ensuring that imported and exported pharmaceuticals were inspected to meet safety and quality standards |
|  | 1 submission suggested **importing medicines from Canada** | 1 submission suggested importing cheaper medicines from Canada as a solution to lowering drug prices |
| Industry (n=20)  American National Standards Institute, ANSI National Accreditation Board, American Chamber of Commerce in Canada, US Chamber of Commerce, National Retail Federation, Aso LLC, World Self-Medication Industry Association, Consumer Health Products Association, Association for Accessible Medicines, US Council for International Business, Canadian American Business Council, Stein Shostak Shostak Pollack & O’Hara LLP, The Borderplex Alliance, Duty Drawback Coalition, Patent Utility Coalition, National Association of Manufacturers, San Antonio Hispanic Chamber of Commerce, National Foreign Trade Council, BIO, PhRMA | 10 submissions discussed **regulatory issues**, including (1) the **harmonization** of Canadian and Mexican **regulatory data protection and approval standards** with those of the United States, (2) the lack of adequate **patent term restoration** mechanisms in Canada and Mexico, and (3) the inclusion of a **Bolar provision** equivalent in the renegotiated agreement | All 10 submissions advocated for greater regulatory harmonization between the United States, Canada, and Mexico. Of these submissions, 9 advocated for the harmonization of Canada & Mexico’s regulatory data protection standards with those of the United states, with 4 specifically urging the adoption of a 12-year regulatory data protection period for biologics and 3 specifically urging Canada’s adoption of a 3-years regulatory data protection period for Rx-to-OTC switches (with the latter 3 arguing that doing so would lower overall healthcare costs by allowing consumers to self-medicate instead of paying for a prescription drug). 1 submission urged officials to prevent Canadian and Mexico regulatory authorities from linking regulatory approval with pricing decisions, in alignment with US and international standards. 1 submission urged officials to harmonize Canadian generic approval standards with those of the United States.  3 submissions expressed dissatisfaction with Canada & Mexico’s patent term restoration mechanisms  1 submission explicitly requested that a Bolar provision (to allow generic/biosimilar manufacturers to use patented inventions during the period of patent term to obtain marketing approval from health authorities) be included in the renegotiated agreement. |
|  | 10 submissions advocated for either **stronger pharmaceutical IPRs** or the **enforcement** of existing pharmaceutical IPRs.  Nearly all of these submissions did so on the rationale that stronger IPRs incentivized greater industry investment and innovation. 1 submission also argued that stronger IPRs allow for the development of drugs that are easier for patients to take and adhere to, resulting in greater long-term costs savings. | 9 submission advocated for strengthened pharmaceutical IPRs. Of these submissions, 4 advocated for strengthened **patent linkage** provisions in Canada and Mexico to prevent the market entry of infringing products, 6 argued that Canada’s patent utility standard for pharmaceuticals was too high (re: ‘**promise doctrine’**), 2 expressed dissatisfaction with how Canadian patent infringement adjudication mechanisms give **preferential right of appeal to generic** companies, and 1 argued that the Canadian standard for disclosing **confidential business information** in the pharmaceutical sector was too low.  1 submission commented generally about the need to enforce patentability standards in the pharmaceutical sector. |
|  | 1 submission advocated for **weaker IPRs** (submitted by a generics/biologics industry association) | Submission expressed opposition to increasing biologic exclusivity periods in Canada and Mexico, and advocated for the inclusion of a ‘paragraph IV 180-day exclusivity’ clause in the renegotiated agreement to incentivize generic challenges to innovator patents. |
|  | 6 discussed issues of **trade and foreign market access**. | 3 submissions advocated for the elimination of tariffs on pharmaceuticals  2 submissions expressed concern about the safety and quality of imported drugs, with 1 explicitly advocating for the prohibition of dumping falsified or substandard pharmaceuticals.  2 submissions discussed the role of the pharmaceutical sector in the American economy. Of these, 1 submission argued that increasing US pharmaceutical exports could serve as a mechanism to close the US trade deficit, while 1 submission highlighted how high-tech industries, including the pharmaceutical sector, have migrated away from the NAFTA region to Asia  1 submission advocated for the general adoption of trade provisions that would enhance market access for US drug makers in Canada and Mexico  1 submission had a specific inquiry re: the export of OTC drugs to Mexico for packaging in first-aid kits |
|  | 7 submissions discussed issues of **ACTA** | 5 submissions underscored the importance of predictability and transparency in the pharmaceutical IPR, regulatory, pricing, and reimbursement space, specifically highlighting the importance of stakeholder participation in the development of rules and regulations pertaining to the biopharmaceutical sector  2 submission urged officials to ensure that foreign regulatory, pricing, and reimbursement systems remain ‘fair and transparent’ to improve domestic industry access to overseas markets.  2 submissions advocated for the inclusion of provisions that improve transparency re: pharmaceutical patents to improve the development of generic medicines. These were submitted by the US Council for International Business and the Association for Accessible Medicine. |
|  | 3 submissions discussed issues in pharmaceutical **pricing and reimbursement** | 2 submissions urged officials to prevent trade partners from artificially lowering the price of medicines (ex through price controls or reference pricing) and advocated for the codification of competitive market-based pricing into the renegotiated agreement  1 submission specifically identified policies that delay the inclusion of new innovative drugs on Mexico’s national formulary as a priority area to be addressed in the renegotiations. |
|  | 5 submissions discussed miscellaneous topics. | 2 submissions discussed specific technical trade provisions (conformity assessments and duty drawbacks) which tangentially affected the pharmaceutical sector  1 submission advocated for the inclusion of a pharmaceuticals/cosmetics/medical devices sector-specific trade subcommittee  1 submission highlighted the importance that NAFTA to continue to be revised as needed in response to new technological breakthroughs in the biopharmaceutical industry  1 submission outlined the importance of the pharmaceutical industry to the economy of its geographic sub-region (Mexico-US Borderplex region) |
| Professional Association (n=5)  United Electrical Radio & Machine Workers of America, International Association of Machinists and Aerospace Workers, Communications Workers of America, AFL-CIO (x2) | 3 submissions broadly sought to **weaken pharmaceutical IPRs** in the renegotiated NAFTA. | 3 submissions urged officials to ensure that IP provisions do not undermine the affordability of medicines, with 2 specifically underscoring a fear that heightened pharmaceuticals IPRs might result in higher drug prices that undermine Medicare/Medicaid programs |
|  | 2 submissions were specifically **opposed to the adoption of TPP terms**. | 1 submission advocated for the rejection of TRIPS+ TPP terms that would strengthen pharmaceuticals IPRs and increase drug prices  1 submission argued that the implementation of TPP provisions would weaken pharmaceutical health and safety standards |
